# Prediabetes: A Benign Intermediate Stage or a Risk Factor in Itself?

**DOI:** 10.7759/cureus.63186

**Published:** 2024-06-26

**Authors:** Irfan G Mulla, Ashish Anjankar, Shilpa Pratinidhi, Sarita V Agrawal, Deepak Gundpatil, Sandip D Lambe

**Affiliations:** 1 Biochemistry, Datta Meghe Institute of Higher Education & Research (DMIHER), Wardha, IND; 2 Biochemistry, Jawaharlal Nehru Medical College, Wardha, IND; 3 Biochemistry, Bharatratna Atalbihari Vajpayee Medical College, Pune, Pune, IND; 4 Biochemistry, Smt Mathurabai Bhausaheb Thorat (SMBT) Institute of Medical Sciences and Research Centre, Nashik, IND

**Keywords:** homeostatic model assessment for insulin resistance (homa-ir), hemoglobin a1c (hba1c), insulin-like growth factor (igf), intermediate, prediabetes risk

## Abstract

Prediabetes is a condition when the blood glucose levels are above the normal range but below the threshold for defining diabetes. Previously considered benign, it is now recognized to be associated with various macrovascular and microvascular complications, with increases in the risk of cardiovascular events, nephropathy neuropathy, and retinopathy. Early identification of prediabetics may help detect the risk for these future complications at an earlier stage. Moreover, therapeutic options for prediabetes are available, which can retard its progression to diabetes and the subsequent development of complications. Hence, we make a case for the early identification of prediabetes through screening methods and appropriate institution of management strategies.

## Introduction and background

When the blood glucose levels are above the normal range but below the threshold for defining diabetes, it is considered prediabetes. Prediabetes is recognized as a predisposing condition for developing diabetes [1]. Prediabetes is not considered benign, and it also has a strong association with microvascular and macrovascular complications [2]. Insulin resistance with subsequent insulin hypersecretion and impaired incretin action is critical in the pathophysiology of prediabetes.

Epidemiology

The prevalence of diabetes is estimated to escalate to 642 million by 2040 from 415 million in 2015 [3]. Most of these diabetic patients are considered to navigate through a stage of prediabetes prior to developing diabetes.

Prediabetes continues to increase globally in prevalence. Estimates state that >470 million are likely to be detected with prediabetes by the year 2030 [1], Around one-tenth of the population in the United States with prediabetes are likely to annually progress to diabetes [4]. The conclusions from a meta-analysis reported the presence of prediabetes at baseline to correlate with increases in the probability of deaths and adverse cardiovascular outcomes. The increased absolute risk for mortality was 7.36 for every 10 thousand person-years and for cardiovascular events was 8.75 for every 10 thousand person-years over a follow-up of 6.6 years [4].

Diagnosis

The criteria for the diagnosis of prediabetes have improved with time but tend to vary among different centers. Prediabetes is diagnosed based on impaired fasting glucose (IFG), impaired glucose tolerance (IGT), and/or increases in glycosylated hemoglobin (HbA1c) [5]. IFG is fasting blood glucose (FBG) between 100 mg/dl (5.6 millimoles per liter) and 125 milligrams per decilitre (6.9 millimoles per liter). However, the World Health Organization (WHO) suggests a narrower range for IFG from 110 to 125 mg/dl [5]. IGT is considered when glucose values are between 140 and 199 mg/dl following the administration of 75 grams of glucose orally (the normal value should be less than 140 mg/dL).

The different screening tests for prediabetes may identify different sets of patients. For instance, some individuals may present with a HbA1c in the prediabetes range, but their fasting blood glucose may be normal. Therefore, if the initial screening test yields normal results but the suspicion of prediabetes in the patient is high, it may be a good idea to confirm with one of the other screening tests, or repeat the test, maybe within one year. Irrespective of the method used for screening, identifying any prediabetic individual prior to the evolution of type 2 diabetes mellitus (T2DM) allows an intervention, such as patient education and preventive measures, or retesting more frequently in the future (for instance, every one to three years).

Trials have previously assessed the frequency of prediabetes in the population. A trial by Sinha and co-workers conducted in the United States of America (USA) depicted that in the age range of 11 and 18 years (adolescents), 21% were found to be obese, with impairments in glucose tolerance, two hours after an oral glucose tolerance test (OGTT) [6]. Another trial by Mohsin and co-workers demonstrated that in the age range of 11 and 18 years, 20% of subjects among the Bangladeshi population had body mass index (BMI) values greater than the 95th percentile for sex and age as per the Centres for Disease Control (CDC) criteria, as well as impairment in glucose tolerance as assessed by the two-hour OGTT [7].

To update, the United States Preventive Services Task Force (USPSTF) conducted a review to reassess the screening data for T2DM and prediabetes in non-pregnant, asymptomatic adults, so as to update its recommendation previously released in 2015 [8]. The population evaluated was between 35 and 70 years of age and was contacted in the primary care settings. Overweight was considered for BMI more than or equal to 25 kilograms per square meter, and obesity for more than or equal to 30 kilograms per square meter. The USPSTF observed a net benefit in screening in that they could offer preventive interventions or refer them for the same. Based on these findings, the USPSTF revised its recommendation that screening should be offered for prediabetics and type 2 diabetics in non-pregnant individuals in the age group of 35 to 70 years with overweight or obesity and suggested that physicians should consider preventive measures for prediabetics or refer them to experts for the same [8].

Studies also suggest that physicians should screen patients for prediabetes at an early age, especially in individuals from areas that have a disproportionately high prevalence and incidence (e.g., Asian Americans, American Indians, Blacks, Native Hawaiian/Pacific Islanders, Hispanic/Latino persons, and Alaskan Natives) or in individuals who provide a history of polycystic ovarian syndrome, a past event of gestational diabetes mellitus (GDM), or a family pattern of diabetes, and lesser BMI values in the Asian American population [9]. Current data hint that a lower BMI of 23 kg/m^2^ might be a more prudent threshold for Asian Americans [10]. Modeling and cohort studies suggest that an appropriate strategy for individuals with glucose in the physiological range may be to screen them every three years [11-13].

## Review

Risks and complications associated with prediabetes

Prediabetes is related to resistance to insulin and malfunctioning of β-cells, both of which generally tend to occur before the glucose abnormalities are detected. Observational evidence has shown that the links of prediabetes are with the initial stages of chronic renal failure, diabetic neuropathy, diabetic retinopathy, diabetic nephropathy, and an elevated probability of cardiovascular complications, which are further exacerbated by an increased risk of atherosclerosis. The employment of multifactorial risk scores may lead to optimization of the detection of the probability for advancement to diabetes with the non-invasive and blood-based (invasive) metabolic parameters. For prediabetics, lifestyle modification is recommended as the standard primary preventive measure, with a relative risk reduction reported to be between 40% and 70%. Data from more recent studies also suggest that pharmacotherapy may potentially benefit these patients [1].

The prediabetic state not only aggravates the potential for T2DM, but it also acts as a clinically important risk for the development of macrovascular disease. Although a part of the macrovascular complication potential can be attributable to the natural progression of the disease to diabetes, an independent concern is considered to persist even in people who do not progress to diabetes [14]. The prediabetic macrovascular complications can be attributed to atherosclerosis, which is also frequently observed in these individuals. Moreover, metabolic syndrome may coexist with prediabetes, which further augments the development of an atheroma. Studies have observed that prediabetics tend to have increases in high-sensitivity C-reactive protein (hs-CRP) and fibrinogen then normoglycemic individuals; both of them are considered to be proatherogenic [15,16].

Individual predispositions for diabetes (e.g., having a close relative with diabetes and a past episode of gestational diabetes) or other risk factors that together constitute the metabolic syndrome (MetS) may also be employed to identify groups at an elevated risk for diabetes; however, their predictive value for the same is found to be inferior to prediabetes. More recently, risk scores are being developed for determining incident diabetes in prediabetic individuals, based on combining both invasive and non-invasive parameters [3].

Cardiovascular diseases (CVDs) and prediabetes

Multiple studies have shown that prediabetes likely exhibits a causal relationship with CVDs and any-cause mortality. As per the conclusions of a meta-analysis of cohort studies performed by Huang and co-workers, an association was concluded between prediabetes and non-fatal stroke, non-fatal coronary diseases, and any-cause mortality [17]. A separate meta-analysis that included 38 prospective studies with mortality or CVD as the end point also concluded that increases in blood glucose tend to be associated with a linear increase in CVDs [18].

Although a direct relationship between coronary vascular disease as a complication and diabetes as a risk factor is widely accepted, many treating physicians remain unaware of the potential risks for the same events with prediabetes. To shed more light on this, a prospective study was performed by Sen et al. on 62 patients presenting with acute coronary syndrome, who underwent admission to a tertiary care hospital in India for management [19]. This study reported that 25% of these patients had diabetes; however, a greater proportion (48.4%) had prediabetes, hinting toward an increase in the risk of the outcomes with prediabetes as well [19].

A study was conducted by the American Diabetes Association (ADA) to determine the relation between prediabetes and atherosclerosis in the coronary arteries [20]. In this study, 67 patients who had experienced an episode of coronary artery disease and had undergone assessment of the main coronary trunk arteries by angioscopy were enrolled. Twenty-three of these patients were found to be diabetic, 28 were prediabetic, and 16 were found to be non-diabetic as per the ADA guidelines. The presence of yellow-colored plaques in the coronary vessels on angioscopy is generally considered the pathognomonic finding for diagnosing acute coronary syndromes, and the same was applied in this study. The occurrence of more than or equal to two such plaques per vessel is interpreted as a predisposition for the occurrence of cardiovascular events in the future. All three groups in this study were evaluated for the number of yellow-colored plaques in each vessel and the yellow-grade intensity. Both the grade and number of the plaques were reported to be significantly more in the prediabetics as against the nondiabetics (p = 0.02 and p = 0.04, respectively), but no such difference was observed between the diabetic and prediabetic groups (p = 0.21 and p = 0.44, respectively). Thus, the authors concluded that both diabetes and prediabetes may act as as independent risks for future cardiovascular events [20].

The association between prediabetes and mean intima-media thickness (IMT) of the common carotids and the calcium in the coronary arteries calcium (CAC) was assessed in the prediabetic and nondiabetic populations in a large study on 272 patients by Scicali et al. [21]. Both the mean IMT and CAC scores were reported to be significantly greater among the prediabetes patients than the non-diabetics (P < 0.001 and P < 0.001, respectively) [21].

Thus, based on the above studies, we may conclude that the state of prediabetes may exert a similar risk on both peripheral and coronary atherosclerosis, as diabetes. However, studies on large-size populations are required to be conducted on the prediabetic population to corroborate these findings.

Although there have been studies, for instance, the Framingham Heart Study, which suggest an epidemiologic relationship between heart failure with diabetes [22], such an established pathophysiologic association has not been hitherto reported for prediabetes in humans. Koncsos et al. have evaluated the association between diastolic heart failure and prediabetes in an experimental animal model [23]. In this study, the researchers induced prediabetes in Long-Evans rats that were fed with a Chow diet high in fats, by injecting streptozotocin in a single dose. The induction of prediabetes was confirmed with impaired insulin and tolerance to glucose, a rise in the fasting plasma glucose, and a rise in the visceral adipose tissue mass. The researchers then assessed the cardiac consequences of these metabolic derangements in this setting of prediabetes. They measured the morphology and functional values in the rat hearts via echocardiography to assess the mass of the left ventricle (LV) and the thickness of the posterior and anterior walls of the LV, all of which were found to be greater in the prediabetes rats than the other group. Other dimensional parameters of the heart on echocardiography were found to be unchanged. The prediabetic rates also showed an increased slope of the graph between the end of diastole pressure and the end of diastole volume of the LV, which is considered an initial, sensitive marker for diastolic dysfunction. On histopathological evaluation, increased mitofusin-2 (MFN2) levels and increased markers of oxidative mitochondrial stress were observed in the vascular smooth muscles. An increased in MFN2 has been documented to correlate with an increase in the induction of apoptosis and cell demise in cardiomyocytes of rats [23].

However, in a study by Essop and co-workers, it was reported that oxidative stress in the mitochondria of the cardiomyocytes of male Wistar rats did not increase even after 16 weeks of induction of prediabetes, indicating that the presence of oxidative stress in the mitochondria may not be realized in all stages and models of prediabetes [24].

The impact of prediabetes on diastolic heart function was studied by Di Pino et al. in 167 patients, whose recruitment was based on HbA1c readings in the range of 5.7-6.4% [25]. In these patients, the researchers reported significantly greater values of sphericity index (SI) (p < 0.05), LA volume (LAV) (p < 0.05), and significantly reduced value of the ratio of E wave (peak inflow through the mitral valve during early diastole) to A wave (atrial filling velocity during late diastolic) (p < 0.05), in comparison with controls. All these parameters, i.e., the SI, LAV, and E/A ratio, are validated early signs of diastolic dysfunction [25].

However, there are many lacunae in the knowledge regarding the most sensitive and specific method to determine with relatively high accurately as to which of the prediabetic patients are more predisposed to develop T2DM in later life. In addition, understanding the pathophysiology specific to various phenotypes may also add value to selecting appropriate screening interventions for these patients and making decisions on their funding. It is also suggested that the management of prediabetes patients should be personalized, preferably by using algorithms or programs that can sufficiently predict the risk specific to the patient’s phenotype and, therefore, allow to tailor the intervention that best fits that patient.

Nephropathy and prediabetes

The available literature is sufficient to establish the link between prediabetes with diabetic nephropathy and chronic renal disease, which are generally identified by tests, such as the estimated glomerular filtration rate (eGFR) and urine albumin excretion rate (AER) [26-30]. The survey conducted by the National Health and Nutrition Examination Survey (NHANES) from 1999 to 2006 revealed that the frequency of albuminuria increased with an increase in blood glucose [29]. Other available data that have studied glomerular filtration rates and increased albuminuria as early markers of renal derangement and involvement in diabetes also corroborate with the concept that changes of nephropathy may already be present, albeit to a limited extent, during the stage of prediabetes itself, before even the proper onset of diabetes [31-33]. By contrast, there is mixed evidence for an association between eGFR, which is a late marker for chronic kidney disease (CKD), and prediabetes, with some of the studies reporting a positive relationship [29], while the other studies do not [30,32]. This may suggest that the changes of nephropathy in prediabetes may be limited to early lesions only. Therefore, longitudinal studies do predict that prediabetes acts as a crucial risk for subsequent complications of nephropathy and CKD. Nevertheless, it is still not clear if this increase in risk is attributable to prediabetes itself, or due to these patients progressing to diabetes, or whether there are any other common causes that contribute to both kidney pathology and hyperglycemia [34,35].

Neuropathy and prediabetes

Neuropathy is further classified into individual subcategories. Autonomic neuropathy is considered to have the strongest association between prediabetes and autonomic; however, the methods used for evaluation have not been optimal. Studies have reported that prediabetes is linked with reduced postural changes in cardiac rate [36] and decreased cardiac rate variability [37], which is regarded to be a marker of parasympathetic function) [37-41], worse performance in the tests for parasympathetic and sympathetic functions [42], and an augmented prevalence of erectile dysfunction among males with prediabetes. However, the evidence that prediabetes is linked with a reduced expiration-to-inspiration ratio, or variations in the cardiac rate while breathing, or with values of orthostatic blood pressure (BP) variations, which is considered a late indicator of diabetic neuropathy, is inconsistent [38].

Studies conducted in prediabetic patients on sensorimotor neuropathy suggest that early diabetic neuropathy with IGT may involve small demyelinated neurons [43-45]. Various tests, such as deep tendon reflexes, the density of distal intraepidermal nerve fibers, total sweat volume, quantitative sudomotor testing, temperature sensation, and arm-to-foot sweat responses, are performed as sensitive markers to detect sensorimotor neuropathy [46,47], whereas other tests, such as classical tests for nerve conduction, perception thresholds for temperature or vibration, the Michigan Neuropathy Screening Instrument, and standardized tuning fork, are reported to be not very useful in capturing neuropathy among prediabetics.

Accumulating evidence also suggests that prediabetes is correlated with a rise in the occurrence of idiopathic polyneuropathy (e.g., small fiber only/sensory neuropathy and idiopathic sensory/painful neuropathy), with IGT more robustly associated with painful neuropathy, than the non-painful ones [48-53].

Retinopathy and prediabetes

An increased risk of diabetic retinopathy may also be present in the prediabetic population, although the evidence supporting this varies based on the techniques used for the detection of retinopathy [54-58]. In a previously reported study conducted on more than 5000 Pima Indians, prediabetes was found to be associated with retinopathy when evaluated with direct ophthalmoscopy [26]. The parameters of retinal vascular changes, such as increased retinal venular or arteriolar caliber and lower arteriole-to-venule ratio, have also been reported to be altered in prediabetes or in patients who presented with an elevated risk of diabetes; however, the supporting literature is inconsistent [56,57,59].

Treatment

All persons of age 40 years and above, and others at a greater risk for diabetes, should undergo regular screening with periodic estimations of HbA1c and/or fasting blood glucose. The goal of treatment for individuals who are diagnosed with prediabetes must be to restore euglycemia, as the currently existing data support that restoration of normoglycemia during the stage of prediabetes or even during the stage of early diabetes may lead to lasting remissions [11].

The recommended approach for this restoration of euglycemia is intensive lifestyle intervention, which has not only shown a reduction in the progression to diabetes but has also shown an overall reduction in mortality due to any cause in longitudinal studies [2]. Intensive lifestyle modification includes increasing physical activities (up to ≥150 min/week), calorie restriction during eating, motivational support, and self-monitoring. When followed rigorously over a three-year period, it has shown a decrease in new-onset diabetes by almost 6.2 cases per 100 person-years [12]. Pharmacotherapy may also contribute to achieving the desired outcomes, as randomized controlled trials (RCTs) with biguanides (metformin), peroxisome proliferation activating receptor (PPAR)-gamma agonists (pioglitazone and rosiglitazone), alpha-glucosidase enzyme inhibitors (acarbose), meglitinides (nateglinide), glucagon-like peptide (GLP)-1 receptor agonists (liraglutide), and pancreatic lipase inhibitors (orlistat) have all shown benefits [2].

Lifestyle interventions

The single most beneficial intervention that tends to reduce the deterioration of prediabetics to diabetes is physical exercise combined with an appropriate diet. One of the earliest studies in support of this was the Diabetes Prevention Study (DPS) conducted in Finland, which was conducted as an RCT enrolling 522 subjects with IGT who were overweight and underwent randomization to receive the standard treatment or an intensive lifestyle intervention [60]. The lifestyle intervention arm in this study received resistance training (circuit-type) and individualized dietary counseling and was advised to intensify their physical activity; on the other hand, the control arm was advised to regularly exercise and have a yearly physical check-up and counseling on a healthy diet. The lifestyle arm was so created that it would undertake a high-intensity activity in Year 1, followed by reduced physical activity in the maintenance period, with the goal being increasing dietary fiber and physical activity, accompanied by decreases in both fat intake in the diet and body weight. The body weights were obtained in the first year and then subsequently at three years. The intervention arm was observed to have lost 4.5 kilos and 3.5 kilos, whereas the control arm lost only 1.0 and 0.9 kilos, respectively. The glycemic and lipid parameters also exhibited better values in the intervention arm, with the intervention arm showing a reduction of 58% in the diabetes risk, in comparison with the control arm. The participants who did not develop diabetes at the termination of the trial period were further evaluated after three more years, and the diabetes incidence, the dietary fiber and fat intake, and their physical activity were measured again [60]. Over the total follow-up of seven years, the authors inferred that the new case onset of T2DM was 7.4 per hundred person-years versus 4.3 per hundred person-years in control and intervention and arms, respectively (p < 0.001), denoting a decrease in the relative risk by 36% [61].

Another large study was the Diabetes Prevention Program (DPP), which reported results akin to the DPS [62]. The DPP study was similar in design, but it additionally included a separate arm treated with metformin that served as a control. The sample size in the DPP study was 3,234 adults at a greater risk for diabetes; out of which, 924 received metformin for treatment, 1,079 underwent intensive lifestyle intervention, and the remaining 932 received a placebo. The intensive lifestyle intervention arm achieved a minimum of 150 minutes of moderate level of physical activity in seven days (which is similar to brisk walking) and a decrease in their body weights by at least 7%. The diabetes incidence was found to be lesser in the lifestyle arm, as well as the metformin arm, compared with the placebo arm (58% and 31%, respectively). The population was analyzed in this study by the intention-to-treat principle, and one case of diabetes was found to be prevented when every 6.9 people were treated [62]. The intensive lifestyle intervention group was further followed up for 10 years, and it was found that for each kilogram of loss of body weight, the risk for diabetes decreased by almost 16% [63]. Thus, the authors observed that the benefits achieved by intensive lifestyle modification on the decrease in the risk for diabetes were maintained for a minimum of 10 years.

Both DPP and the Finnish studies successfully demonstrated that intensive modifications in lifestyle can benefit a long way in the management of prediabetes. Nevertheless, a major challenge still persists in implementing the intervention successfully in the community. Katula and co-workers applied the intervention studied in the DPP at the level of the local community [64]. In this study, community healthcare workers were instructed to administer a regimen of lifestyle weight loss (LWL) as a part of a community education program to a group of people in the community. The community healthcare workers were chosen as those who had a healthy lifestyle and/or well-controlled diabetes so that they themselves could serve as role models to the community. This study enrolled 301 prediabetic obese volunteers. The lifestyle weight loss intervention set two valuable goals for the study subjects: to attain a minimum of 180 minutes of physical activity in seven days and to restrict their consumption of calories to 1,200 and 1,800 kcal per day [64]. These goals were set in accordance with the recommendations of the American Society of Clinical Nutrition, the North American Association for the Study of Obesity, and the American Diabetes Association (ADA) [65]. The study participants attended almost two-thirds of all the conducted intervention sessions. The study participants were found to attain a weight reduction of approximately 6% and a decrease in the waist circumference of almost 5%, over 12 months, when compared with those who received the standard treatment. The FBG also reduced by 4.3 milligrams per deciliter in the LWL arm, in comparison with a reduction of only 0.4 milligrams per deciliter in the standard treatment arm (p < 0.001) [65].

Pharmacologic approaches

Currently, there are four pharmacological options to treat the prediabetic population, i.e., metformin, acarbose, pioglitazone, and liraglutide. In addition, three pharmacological therapies for weight loss have also been proposed by the Association of American Clinical Endocrinologists (AACE), i.e., orlistat, phentermine + topiramate extended-release (ER), and lorcaserin, for the management of obesity, with the goals of treatment being reversal of resistance to insulin and halting the deterioration to T2DM [66].

The most supporting evidence for the medical management of prediabetes is with metformin. Other medications that have been found to be efficacious are alpha-glucosidase inhibitors, thiazolidinediones, basal insulin, orlistat, and valsartan. However, none of them have been so far recommended for this purpose [11].

Metformin has shown a benefit in reducing the potential for diabetes among prediabetic individuals by around three cases for every hundred person-years over a three-year duration. The medication has been found to be most effective in individuals below 60 years of age having a fasting blood glucose of above 110 mg/dL, or HbA1c of above 6.0%, or BMI of ≥35 and in females with past gestational diabetes [12].

Metformin acts by reducing the hepatic production of glucose by impeding the respiration in the mitochondria of the hepatocytes, leading to a subsequent activation of 5′ adenosine monophosphate-activated protein kinase (AMPK), an intracellular enzyme that performs a crucial part in homeostasis within the cell). This increases the peripheral tissue sensitivity to insulin and lowers cyclic AMP (cAMP), which decreases the expression of the enzymes involved in gluconeogenesis. There are also effects attributed to metformin independent of AMPK activation, such as inhibition of the enzyme fructose-1,6- bisphosphatase. A meta-analysis performed by Salpeter and co-workers evaluated data on 4,560 participants [67]. The incidence of diabetes was found to reduce following metformin treatment by 40% (odds ratio (OR) = 0.6; 95% confidence intervals (CI) = 0.5-0.8), with a reduction in absolute risk by 6% (95% CI from 4 to 8) over a follow-up period of almost two years [67]. In another study by Ramachandran et al. conducted on 531 native Asian Indians, both metformin (26.4%) and lifestyle intervention (28.5%) were found to decrease the potential of T2DM, in comparison with control [68].

In another study by Svensson et al., the relationship between reduction in HbA1c on metformin therapy and the incidences of mortality and cardiovascular outcomes was assessed in T2DM patients [69]. This study recruited 24,752 patients started on metformin. Over a follow-up of two and a half years, the incidence of mortality and cardiovascular events observed in participants who managed to achieve HbA1c levels of less than 6.5% was lower during the six months of initiating metformin [69]. However, more studies need to be conducted before applying these results to the population of prediabetes, nevertheless, a potential for cardioprotection with metformin cannot be ruled out at this stage.

Pioglitazone, a thiazolidinedione, serves as a ligand on the peroxisome proliferator-activated receptor-gamma (PPAR-gamma), which results in an alteration of gene transcription, which plays critical parts in the metabolism of carbohydrates and lipids, with a resultant enhancement in insulin sensitivity. The additional benefits attributed to pioglitazone include enhanced insulin signaling and a rise in the expression of glucose transporter (GLUT)1 and GLUT4 (transporters of glucose). Defronzo et al. conducted the ACT NOW trial, in which 602 participants were randomized to receive either placebo or pioglitazone 30 mg every 24 hours (which was increased to 45 mg every 24 hours after the first month) [70]. After a duration of two and a half years, a lesser tendency of developing diabetes was reported, compared with placebo, for pioglitazone (2.1% vs. 7.6%) [70]. Pioglitazone also showed reduced risks of ischemic events and stroke in the IRIS trail [71]. The initial stroke event rate was found to be 9% with the treatment (pioglitazone) group versus 11.8% with the control groups [71]. In addition, the incidence of T2DM was also lower with pioglitazone (3.8%) against the placebo (7.7%) [71]. The drug appears to be relatively safe in the prediabetic population, so long as they do not have any baseline comorbidity that increases their risk of complications from weight gain or bone fractures. The common adverse effects reported with pioglitazone include an increase in weight of ≥4.5 kg, an increase in pathological bone fractures, and edema.

Acarbose is an alpha-glucosidase inhibitor and is found to be maximally active against the enzyme glucoamylase, followed by others, such as maltase, sucrase, and dextranase. It delays the breakdown and hence absorption of sugars in the large intestine. Chiasson and co-workers performed an RCT, in which 1,429 participants were randomized to placebo (n = 715) or acarbose (100 mg every eight hours) (n = 714) [72]. The incidence of new-onset diabetes was found to be 32% with acarbose, as against 42% with placebo (p = 0.0015), with a reported number needed to treat (NNT) of 10. However, the adverse effects experienced with acarbose included diarrhea and flatulence, which led to early discontinuation of acarbose in the test group [72]. The participants were further followed up by the author who observed that the potential for cardiovascular events in participants with IGT in the long term was also reduced with acarbose (2.2%) when compared with placebo (4.7%) (the risk for developing myocardial infarction (MI) was 0.15% vs. 1.75% for acarbose and placebo). However, the differences in placebo and acarbose were found to be similar in other parameters [72].

Liraglutide is a mimic of the GLP-1 and acts as a GLP-1 agonist with biochemical features similar to GLP-1. The most significant effect of liraglutide therapy is a rise in the secretion of insulin from the beta cells of the pancreas as a response to oral carbohydrate ingestion (secretagogue action), which is also accompanied by the suppression of secretion of glucagon, decreased food consumption, slowed gastric emptying, and proliferation of beta-cells. The SCALE Obesity and Prediabetes trial was an RCT conducted on 2,254 prediabetic individuals who presented with a baseline BMI of 30 kilograms per square meter or more or 27 kilograms per square meter or more with other comorbidities [73]. After a follow-up of 160 weeks, liraglutide showed a lessening in the potential of diabetes as against the placebo arm (2% vs. 6%; p < 0.0001) with an NNT of 25. Liraglutide also showed a greater weight loss (6.1% vs. 1.9%; p < 0.0001), an increased proportion of normoglycemia (66% vs. 36%; p < 0.0001) with an NNT of 4), and a similar rate of side effects (15% vs. 13%) with the placebo group. The withdrawal rate was also found to be significantly high in both groups (47% with liraglutide, 55% with placebo) [73]. A cardioprotective effect has been attributed to liraglutide, but this is yet to be properly evaluated in prediabetics. The LEADER trial has shown a lessening in cardiovascular events with liraglutide after a follow-up of 3.8 years in T2DM patients [74]. The primary end point of this study was a composite of cardiovascular mortality, non-fatal stroke, or non-fatal MI. It was significantly lesser in the liraglutide arm (13.0%) as against the control (14.9 percent) (hazard ratio (HR) = 0.87; 95% CI = 0.78-0.97; p = 0.01 for superiority, and p = 0.001 for noninferiority) [74]. The authors, therefore, concluded that liraglutide exhibits sufficient potential in prediabetes management and may also lead to an improvement in cardiovascular health.

Orlistat, a semi-synthetic derivative of lipostatin, is a selective and potent antagonist of both gastric lipase and pancreatic lipase. The drug binds to the serine amino acid moiety of the lipase enzyme and inhibits triglyceride hydrolysis, subsequently decreasing the intestinal absorption of free fatty acids and monoacylglycerides. In an RCT by Torgerson et al., 3,305 individuals with BMI 30 kg/m^2^ or greater having either normal tolerance to glucose or IGT were enrolled and randomized to receive orlistat + lifestyle intervention 120 mg every eight hours or lifestyle + placebo [75]. After a follow-up duration of four years, orlistat showed a significant decrease in the occurrence of T2DM (6.2%) as against placebo (9%) (p = 0.0032) with an NNT of 36. It also showed a significantly greater weight loss (mean = 5.8 kg) versus placebo (mean = 3 kg) (p < 0.001) and reduced the deterioration to IGT from normal (27.6% vs. 30.5%). However, the sharpest criticism of the study was the huge rate of dropouts (48% with orlistat vs. 66% with placebo); nevertheless, the analysis in 99% of the participants was conducted on an intention-to-treat basis [75]. To date, studies have not been performed to determine any cardioprotection with orlistat.

Phentermine, an alpha agonist, is a centrally acting suppressant of the appetite center that increases metabolism via its agonistic action on alpha-1 receptors, and topiramate, an anti-epileptic, has a supposed mechanism of suppressing appetite and enhancement of satiety through the involvement of neurotransmitters. Guo et al. performed a study on 3,040 participants, in which data were pooled by the authors from three RCTs (SEQUEL, EQUIP, and CONQUER) [76]. The participants who were found to be obese or overweight but without diabetes were administered 15 milligrams/92 milligrams or 7.5 milligrams/46 milligrams once daily, respectively, of phentermine/topiramate and assessed against a placebo control group for more than one year. The patients were also stratified for their risk for developing diabetes with the Cardiometabolic Disease Staging score. The authors observed that the occurrence of diabetes was 0.67%, 2.37%, and 6.29%, for the minimum, intermediate, and maximum risk groups, respectively, on topiramate, significantly lesser than that for placebo (1.51%, 4.67%, and 10.43%, respectively) [76]. However, the potential cardioprotective benefits of phentermine/topiramate have not been evaluated to date. In addition, there are teratogenic concerns and tachycardias reported with phentermine.

Lorcaserin is a small-molecule agonist at the serotonin receptors in the central NS, primarily in the hypothalamus, where it can suppress the appetite. It has been positioned for promoting a reduction in weight in overweight or obese, as an add-on therapy to increased physical activity and low-calorie diet. A post-hoc analysis has been performed by Nesto et al. by pooling data from two phase III studies (Bloom and Blossom) with a total sample size of 6136, with an aim to monitor weight and glucose parameters for duration of 52 weeks in the overweight or obese prediabetics [77]. The authors observed that 3.2% of the prediabetic patients on lorcaserin progressed to T2DM, while the corresponding proportion was significantly higher with the placebo group at 5.0% (p = 0.032). Interestingly, the reduction in the progression to T2DM was statistically significant when evaluated with the HbA1c but not significant when evaluated with the fasting blood glucose. In addition, the reversion to euglycemia was also significantly greater with lorcaserin, when evaluated both with HbA1c (40% vs. 29.5%, p < 0.001), as well as fasting plasma glucose (52.4% vs. 46.5%, p = 0.047) [77]. Similar to other drugs, no studies have evaluated any potential cardioprotective benefits of lorcaserin.

Other approaches

Surgical measures such as bariatric surgery have been found to be beneficial in preventing T2DM in obesity and prediabetes [2]. Bariatric surgery is an option in obese persons with a BMI of 30-35 with concomitant metabolic syndrome [11].

It is recommended that among the obese (with a BMI of 40 or above) who are considered eligible candidates to undergo bariatric surgery, those with prediabetes must be given priority, as they are at the maximum probability for diabetes and its subsequent sequelae, which may be most benefitted with the reduction in weight caused by the surgery [78]. This recommendation is supported by a post-study analysis of participants in the Swedish Obese Subjects study on 4,032 obese participants, out of whom, half underwent bariatric surgery while the other half received the standard care. Following 15 years, those in the prediabetes, diabetes, and normoglycemia groups who received bariatric surgery experienced a larger reduction in macrovascular complications; the greatest reduction was observed among the prediabetics [79].

## Conclusions

Thus, based on our review, we conclude that prediabetes is not a benign condition, but rather a risk factor in itself for progression to diabetes, as well as the subsequent development of complications. Moreover, therapeutic options for prediabetes are available, which can address the issue at the early stages. Hence, we make a case for early screening for prediabetes, especially among those with high risk, such as the presence of family history, presence of hyperlipidemia, obesity, and other predisposing conditions, so that appropriate management strategies can be instituted and the complications can be prevented. Nevertheless, whether such interventions actually reduce the risk of complications is something that does not have any evidence as yet and therefore requires further study.
